# Faithfulness and Kingship

**Published:** 1892-11-19

**Authors:** 


### The Hospital

AN INSTITUTIONAL JOURNAL OF

Science, Medicine, Nursing, and Philanthropy.

*For Hospitals, Asylums, Infirmaries, Dispensaries, Medical Practitioners, Students, Nurses, and the Charitable Public.*

VOL. XIII.—No. 321.] [Nov. 19, 1892.

### FAITHFULNESS AND KINGSHIP.

It would not have been anything but fitting if some venerable minister of religion had pronounced one word of eulogy over the coffined body of John Charles Steele, in the quadrangle of Guy's Hospital last week, as he was carried out to his funeral. There, in the presence of the hospital authorities, of the grey-haired senior physician and all the other physicians and surgeons, of the senior and junior medical students, the matron, the sisters, and the nurses, of a great number of representatives of other hospitals, and of private friends of the deceased, above all in the presence of that large crowd of sorrowing poor from the surrounding district who had flocked so eagerly to pay a last tribute of respect and love to one who had been their friend in many an hour of sickness for forty years, it would have been fitting that the voice of authority and of religion should have pronounced a modest "Well done." The true epitaph of the dead man might have been uttered in a sentence: "He did his work well; he did it all his life; he died at his post: *Si monumentum quaeris circumspice*."

Dr. Steele belonged to a class of persons who, when they die, though they are not "unwept," nor even "unhonoured," are certainly "unsung." He was conspicuous in life for one thing, and perhaps for one thing only. He did the work which was his allotted task, and did it always well. It was not an insignificant post to which he was appointed when he became medical superintendent of Guy's Hospital. He was the chief minister, perhaps the king, in a peculiar kingdom—the kingdom of the sick and the injured, the feeble and the defenceless. His subjects were many, but they all had this in common, that they needed a human providence to take entire charge of them, to heal their sicknesses, and to minister to their smallest as well as to their greatest wants. A still greater company of occasional subjects, in the shape of out-patients, looked to Guy's Hospital and to Dr. Steele in the hour of their need and distress. A daily task of great magnitude and of supreme importance, it is evident, was the work of this man. How did he acquit himself in it? His power for good was unlimited, and his power for evil hardly less so. He ruled the little kingdom of Guy's Hospital for nearly forty years, and when he died it was almost as if the hospital itself had come to a temporary standstill. Nobody has acclaimed the dead man in the public press; and yet he was a rare man, and deserved to have been acclaimed.

Dr. Steele was not of those whose work is in public places, and who, therefore, become conspicuous among their fellow-men, even though their own peculiar merits be sometimes of the smallest. But he belonged to that absolutely indispensable class whose place is in the very centre and stress of the actual work which the world is daily doing. Little that he did was "seen of men." But all that he did was known to a discriminating few in the hospital world, and to large numbers of the South London poor who, to the great world, are not so much as names or even numbers. The discriminating few laid him to his rest with true mourning; and some of them felt with a pang of half-remorseful surprise that they were burying a man whose life and work had been altogether greater than they had given him credit for. But the nameless poor who flocked into the hospital quadrangle felt no such pang of surprise and half-remorse. They had always known that Dr. Steele was a kindly and helpful man; that he made Guy's Hospital a welcoming home for them when sickness and helplessness pressed them hardly. They knew that he was not too stern, not too stiffly official; he was human, and fatherly, and had a touch of wholesome humour about him. The women and children of South London loved this friendly soul, and so, too, did the men. He shone like a kindly light in a place where not only the night but the day is often gloomy; and for all these things the poor, with one voice, blessed him.

But Dr. Steele found a way to other hearts as well as those of the poor. Nothing at all but the truest worth can win the respect and affection of those very peculiar human beings who constitute the medical and surgical staff of a large London hospital. The life-work of such men is so full of stern reality that unreality of any kind becomes painful and almost hateful to them. This medical and surgical staff held Steele not in regard only, but in affection. The old man was dear to them. It was the same with the students, senior and junior, industrious and "chronic." They had all learnt to trust him, they knew he understood them, and would take trouble to serve them. But perhaps the regard which rises to enthusiasm was most conspicuous among the sisters and nurses of Guy's Hospital. Dr. Steele felt like a father towards them all, and they all in turn learnt to give him their confidence and to seek for his helpful guidance exactly as they would that of a father; and all this for nearly forty years. Generation after generation of students came and went; probationer nurses became sisters, and sisters matrons; even the members of the medical staff commenced their career, became mature, and grew old with Steele still there in his old place through all the changeful years. It was as if he had been born with the hospital, and was its living soul, without which it must almost cease to be Guy's.

Now all this, we contend, is worth thinking of. We invite hospital workers of every class to think of it. A truly honourable life was laid down when Dr. Steele died. He painted no great picture, he wrote no great book; but if ever the deeds of men are weighed up, Dr. Steele will have a place amongst those true royalties who, through a long lifetime, have been the faithful and loyal servants of the subjects committed to their charge.

It is not our wish to preach a funeral sermon, but it is our desire to place before hospital workers of every class the greatness of the work in which they are engaged, the noble nature of the opportunities which lie before them, and the high esteem and honour they may win from a discriminating few—and, shall we not say, from a still higher and more supremely-righteous judge? By doing loyally, and with their whole heart, the work which lies to their hands in hospital life, they may win the verdict from their fellow-men that they are not unworthy of immortality. After all, the doctor and the hospital worker has no natural clinging to the creed of the materialist. Hospital life does not abolish humanity in men or women; rather it makes them more truly and tenderly human. Their work and their duty are almost entirely hidden from the great world; and, however marvellous and even brilliant the work may sometimes be, they seldom receive the plaudits of the admiring crowd. But in his heart of hearts the deep-thinking and sober hospital worker, though beset by many doubts unknown to those who think less than he, admits, perhaps timidly, the hope that one clear eye may see his work, that one unerring mind may judge it, and that at some future day, in the long life of the universe, his wholesouled efforts of mind and will may be placed side by side with the greatest of the great public achievements that have won the admiration of all who have seen them in the world of time. In the long run it is our hope that kingdoms will be to the kingly. Dr. Steele has had his little kingdom, and has administered it well to the end. Why should not such fidelity receive a still higher reward? In the most venerable of books we are taught that those who are faithful and loyal to high duty to the end of life shall be made "kings and priests, and shall reign for ever and ever." Can there be any higher duty than that of the hospital worker? Can any man be worthier of a glorious immortality?

